# Severe Hypercalcemia in an Adolescent with New-Onset Diabetes Mellitus and Diabetic Ketoacidosis

**DOI:** 10.7759/cureus.8435

**Published:** 2020-06-04

**Authors:** Shilpa Gurnurkar, Emily R DiLillo, Mauri Carakushansky

**Affiliations:** 1 Pediatric Endocrinology, Nemours Children's Hospital, Orlando, USA; 2 Pediatrics, Nemours Children's Hospital, Orlando, USA

**Keywords:** pediatric diabetes, severe diabetic ketoacidosis, hypercalcemia, hypernatremia, dehydration

## Abstract

Severe hypercalcemia in diabetic ketoacidosis (DKA) among children is rare and can be life-threatening. Its exact etiology is not clear and several mechanisms related to dehydration and metabolic acidosis have been proposed. Rigorous hydration with the correct fluid choice usually corrects the hypercalcemia in those without other underlying causes of hypercalcemia such as hyperparathyroidism. Specific medications to treat the hypercalcemia may be avoided. We present a 13-year-old girl with new type 1 onset diabetes mellitus in DKA with unusually severe and persistent hypercalcemia and severe hypernatremia that gradually responded to rigorous intravenous hydration with Plasmalyte A (Baxter International Inc., Deerfield, Illinois).

## Introduction

Approximately 30% of youth present with diabetic ketoacidosis (DKA) at the time of the diagnosis of type 1 diabetes mellitus [1]. Various electrolyte abnormalities, such as hyponatremia, hypernatremia, hyperkalemia, and hypokalemia, may be seen in patients with DKA [2]. However, severe hypercalcemia in diabetic ketoacidosis is rare, especially in the pediatric population, with very few reports in the literature [3]. Various modalities to treat significant and unexpected hypercalcemia have been proposed, including vigorous hydration, diuretic therapy, as well as bisphosphonate infusion [3]. With regard to sodium abnormalities, mild hypernatremia can be seen in approximately 30% of DKA cases with significant hypernatremia being a rare event [4]. Typically, adequate hydration sufficiently corrects the hypernatremia. We present a case of an adolescent girl with new-onset diabetes mellitus, who presented in severe DKA and had significant hypercalcemia and hypernatremia that responded well to vigorous and prolonged hydration alone.

## Case presentation

A 13-year-old female with no significant past medical history and negative family history for diabetes mellitus presented to an outside facility with altered mental status and was found to be in DKA associated with new-onset type 1 diabetes mellitus.

In the two months leading up to her hospitalization, she reportedly had a significant weight loss and had complained of increased urination and increased thirst. She traveled to our area a few weeks prior to presentation to visit family, and during that period, she began sleeping excessively and complained of abdominal pain and nausea. On the day of hospital admission, she was noted to be disoriented and soon became unresponsive for which emergency services were called.

When emergency services arrived, she was noted to have altered mental status, and her blood glucose was over 650 mg/dL. Upon arrival to the emergency room at the outside facility, venous blood gas indicated a pH of 6.9 (normal range 7.34-7.43), bicarbonate of 2 mmol/L (normal range 22.0-26.0 mmol/L), glucose over 700 mg/dL (normal range 54-117 mg/dL), and large urinary ketones, consistent with severe DKA. Additionally, she had significant hypernatremia and elevated ionized calcium level, with corrected serum sodium of 160 mmol/L (normal range 134-143 mmol/L) and ionized calcium of 1.6 mmol/L (normal range 0.97-1.30 mmol/L).

In the emergency room, she was treated with a 1-liter fluid bolus of lactated Ringer’s solution and a 10-unit intravenous regular insulin bolus. She was then transferred to our pediatric intensive care unit, where assessment indicated a Glasgow Coma Scale (GCS) score of 9 and significant Kussmaul breathing requiring up to 2 liters of oxygen via nasal cannula. Repeat labs were obtained and indicated a pH of 7.04, bicarbonate of 2.5 mmol/L, glucose of 488 mg/dL, corrected serum sodium of 166 mmol/L, ionized calcium of 1.86 mmol/L (serum calcium 12.1 mg/dL - normal range 8.8-10.6 mg/dL), blood urea nitrogen of 15 mg/dl (normal range 7-17 mg/dl), and serum creatinine of 1.1 mg/dl (0.45-0.81 mg/dl). Of note, her hematocrit was 48% at this time, suggesting hemoconcentration.

She was immediately started on an insulin drip with regular insulin at 0.1 units/kg/hour and intravenous (IV) fluids using Plasmalyte A (Baxter International Inc., Deerfield, Illinois) at 120% of her maintenance rate upon arrival.

About four hours after admission, her labs revealed minimal changes, with a pH of 7.08, bicarbonate of 6.0 mmol/L, and glucose of 408 mg/dL. IV fluids were then increased to 150% of the maintenance rate. Notably, her corrected serum sodium level was 163 mmol/L, and her ionized calcium was 1.81 mmol/L. The highest ionized calcium level was 1.92 mmol/L on hospital day one. Her initial serum phosphorus level was low at 2.2 mg/dl and normalized eight hours after arrival at our hospital. On the other hand, her ionized calcium levels remained elevated despite improvement in the pH and continued hydration. Intact parathyroid hormone (iPTH) level was appropriately suppressed for the degree of documented hypercalcemia at less than 3.4 pg/mL (normal range 10-65 pg/mL) when the ionized calcium was 1.83 mmol/L. The alkaline phosphatase level was normal at 282 U/L. Medications to treat hypercalcemia such as calcitonin and bisphosphonates were at some point entertained but a decision was made to target her significant asymptomatic hypercalcemia primarily with aggressive hydration. Other relevant labs included a normal serum creatinine kinase (ruling out rhabdomyolysis) and normal amylase and lipase levels (ruling out pancreatitis). Hemoglobin A1C was 13%, confirming long-standing hyperglycemia.

**Figure 1:**
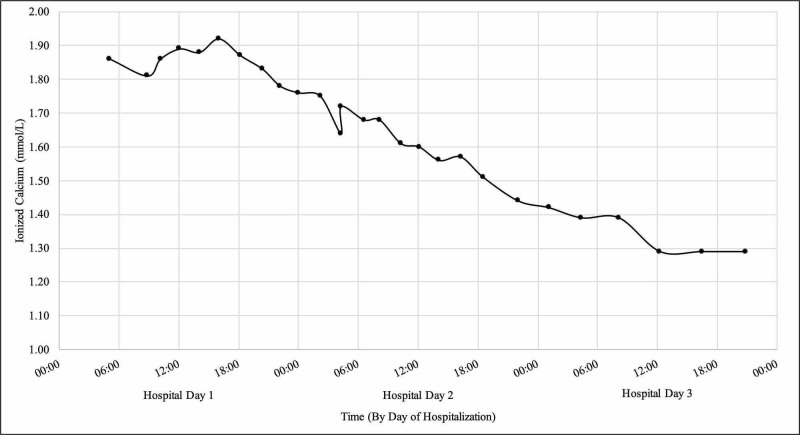
Trend in ionized calcium level during hospital stay

About 24 hours after admission, the DKA resolved, with improvement in both mental and respiratory status. Her pH was 7.32, bicarbonate was 21.7 mmol/L, and serum glucose was 175 mg/dL. However, corrected serum sodium at that time remained significantly elevated at 157 mmol/L while ionized calcium also remained high at 1.72 mmol/L (total calcium of 11.9 mg/dL). The patient continued receiving insulin infusion due to the persistent electrolyte derangements and the inability to tolerate a clear liquid diet. Fifty-five hours after admission, her ionized calcium corrected to 1.29 mmol/L (total calcium 9.5 mg/dL), but her corrected sodium level was still significantly elevated at 162 mmol/L (Figure 1).

**Figure 2:**
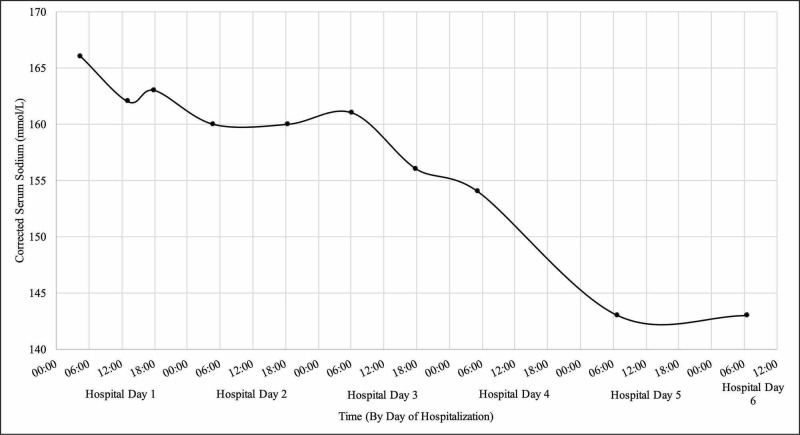
Trend in corrected serum sodium level during hospital stay

Roughly 60 hours after admission, she had persistently elevated serum sodium levels with corrected sodium of 161 mmol/L despite a normal hematocrit of 33%. Therefore, her IV fluids were changed to 180% of the maintenance rate. The next morning, her mentation returned to baseline and she was transitioned to a regular diet and started on a subcutaneous insulin regimen. Intravenous fluids were stopped once serum sodium was lower than 150 mmol/L. Ninety-eight hours after admission, her serum sodium entered the normal range with corrected serum sodium of 143 mmol/L (Figure 2).

The patient also had a head computed tomography (CT) scan and chest X-ray performed in the emergency room. The CT scan was normal.

**Figure 3:**
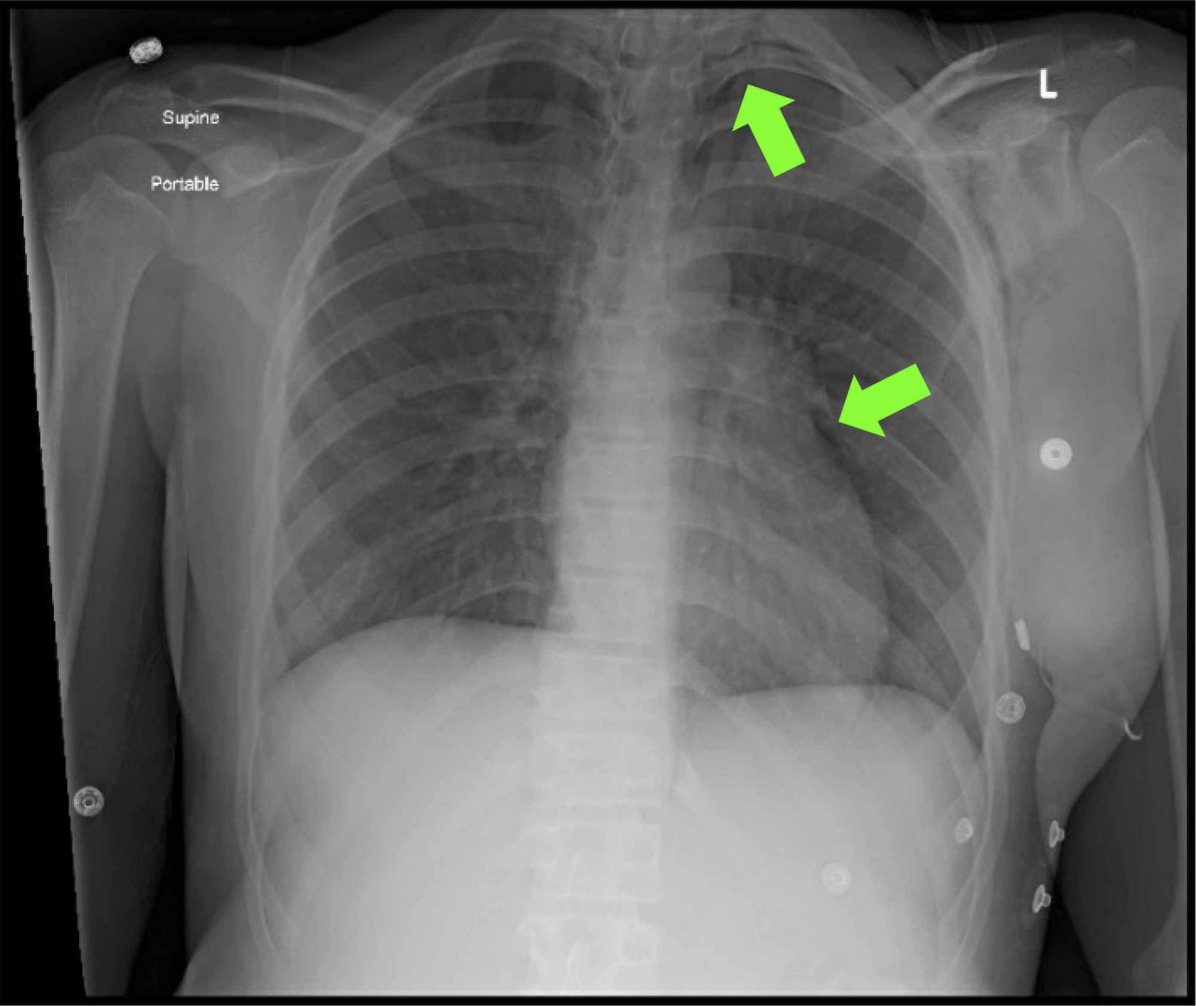
Initial chest X-ray showing pneumothorax and pneumomediastinum

Her chest X-ray, however, showed a small left apical pneumothorax and a pneumomediastinum, which were thought to be secondary to her Kussmaul breathing (Figure 3). She initially required oxygen to maintain normal saturations but was able to be weaned once her acidosis corrected. Repeat chest X-ray at discharge indicated near resolution of both pneumothorax and pneumomediastinum.

## Discussion

DKA is a state of profound insulin deficiency, characterized by hyperglycemia, intravascular volume depletion, as well as metabolic acidosis. A significant percentage (30%) of children and adolescents with newly diagnosed type 1 diabetes mellitus present in DKA [1]. In general, the most common cause of hypercalcemia in patients with diabetes mellitus is hyperparathyroidism, which our patient did not have [5]. Significant hypercalcemia in patients with DKA who do not have hyperparathyroidism is a rare event. To the best of our knowledge, there is only one published report in the pediatric age group [3]. Additionally, in a study published by Topaloglu et al., the average ionized calcium measured in children with new-onset type 1 diabetes presenting in DKA was only minimally elevated at 1.37 mmol/L while our patient presented with far more pronounced hypercalcemia [6].

Various mechanisms for hypercalcemia in DKA have been hypothesized. Metabolic acidosis has long been known to induce the efflux of calcium from the bones via increased osteoclastic bone resorption and decreased osteoblastic bone formation [7]. Additionally, in mice, one study has shown that elevated glucose level results in decreased calcium uptake into the bone [8]. Most of the data, however, is from in vitro studies. Topaloglu et al. demonstrated that patients presenting in DKA tend to have depression of osteoblastic markers such as plasma osteocalcin level and serum alkaline phosphatase activity. These changes reflect impaired bone formation during a state of metabolic acidosis. The fact that osteoblastic parameters normalized with the correction of acidosis suggests that metabolic acidosis was likely the main factor leading to decreased osteoblastic activity and, potentially, the hypercalcemia in DKA [6]. Dehydration, secondary to hyperglycemia, osmotic diuresis, and poor oral intake, has been proposed to exacerbate hypercalcemia [9]. Dehydration, even in the absence of hyperglycemia, has been reported to cause hypercalcemia [10]. Rhabdomyolysis, which is occasionally seen in DKA patients, may also lead to hypercalcemia [11]. Our patient, however, had a normal serum creatinine kinase level, ruling out muscle breakdown as the primary cause of the hypercalcemia.

Because the proposed mechanism of hypercalcemia in DKA is largely dehydration and metabolic acidosis, rigorous hydration is the first-line treatment in correcting hypercalcemia. Additional treatment options, such as calcitonin and bisphosphonate therapy, are rarely necessary if hypercalcemia is purely secondary to the DKA. Our patient required large amounts of IV fluids at 150% of the maintenance rate for the hypercalcemia and hypernatremia to eventually improve and such management allowed us to avoid the use of specific medications to target hypercalcemia.

Both hyponatremia and hypernatremia may be observed in patients with DKA, with hyponatremia being more common [2,4]. Our patient had significant hypernatremia with a corrected serum sodium level of 160 mmol/L at the initial presentation with the level increasing to as high as 166 mmol/L on day one of the intensive care unit (ICU) stay. Similar to hypercalcemia, the exact mechanism of severe hypernatremia is not clear. Dehydration is likely a major contributing factor. Additionally, Monroe and group proposed that in patients with new-onset diabetes mellitus, the negative feedback of hyperinsulinemia leading to increased proximal renal tubular sodium reabsorption may be exceptionally efficient, thus resulting in sodium retention [4]. The authors also noted that hypernatremia in patients with new-onset diabetes mellitus was more commonly seen among the most seriously ill individuals. Our patient’s hypernatremia gradually responded to hydration with isotonic fluids. Plasmalyte was the IV fluid of choice in this patient. Plasmalyte is most similar to plasma in its physiologic content with 140 mEq/L of sodium, 5 mEq/L of potassium, and 98 mEq of chloride. In comparison, 0.9% normal saline contains 154 mEq of sodium, 154 mEq of chloride, and no potassium. Her serum sodium level returned to the normal range 98 hours after admission.

## Conclusions

Electrolyte derangements are common among patients presenting in DKA. Severe hypercalcemia in DKA, however, is rarely seen in children, with only one previously reported case. It is important for intensivists to be aware of less frequently documented electrolyte derangements that could be associated with DKA and of the appropriate intravenous fluid management required to safely correct the observed electrolyte abnormalities. In doing so, unnecessary use of specific medications to treat hypercalcemia may be avoided.
